# Poplar Hospital for Accidents

**Published:** 1895-01-12

**Authors:** 


### POPLAR HOSPITAL FOR ACCIDENTS.

Sir,—In your issue of January 5th you have a drawing of a child's cot. I have taken a great deal of trouble to secure the best one for this hospital, and the one we have is very like that in use at the London. It is better than the one you have a drawing of, because the side which slides down is fixed to the "posts," and so the whole side of the cot lets down and there is no projection to catch the "funny bone" or tear the nurses' dresses. I do not want to use your paper as a means of advertising any firm, so do not give the names of the makers. Anyone interested can learn at the hospital.

SYDNEY HOLLAND.

[We propose to give a drawing of the cot in use at the Poplar Hospital as soon as possible for the benefit of our readers.—ED. *T. H.*]

